# How much does a forensic autopsy cost in Spain?

**DOI:** 10.1007/s12024-022-00534-w

**Published:** 2022-11-07

**Authors:** Fernando Martín-Cazorla, Valentín Ramos-Medina, Leticia Rubio-Lamia, Ignacio Santos-Amaya, Francisco Jódar-Sánchez

**Affiliations:** 1 Forensic Pathology Service, Institute of Legal Medicine, Málaga, Spain; 2 Legal and Forensic Medicine Unit, Department of Human Anatomy, Legal Medicine and History of Science, School of Medicine, University of Málaga, Málaga, Spain; 3 Málaga Biomedical Investigation Institute - IBIMA, Málaga, Spain; 4 Department of Statistics and Econometrics, University of Málaga, Málaga, Spain

**Keywords:** Forensic autopsy, Cost, Health economics, Forensic medicine

## Abstract

**Introduction and objectives:**

The autopsy is considered the gold standard in death investigation. Performing an autopsy requires human and material resources that must be programmed in order to meet the demands of the judicial system. However, as far as we know, the cost of forensic autopsy in Spain has not been determined. Thus, the aim of this study was to estimate the cost of a standard autopsy in order to organise Forensic Pathology Services more efficiently.

**Material and methods:**

A micro-cost analysis was carried out. The nominal group technique was applied using a panel of 10 forensic experts in order to identify and quantify the resources associated with a forensic autopsy.

**Results:**

The results showed that analysis and studies are the most important item in the total cost (54.7%), followed by staff (20.5%), preservation of body (14%), single-use products (7%), equipment and stock (1.6%), cleaning and disinfection (1.5%), facilities maintenance (0.5%) and IT (0.2%).

**Conclusions:**

The total cost of a standard autopsy was €1501.45, which is lower than the European average. This study is the first in Spain to calculate the unit price of a forensic autopsy by means of micro-cost analysis. This may help to address the way forensic pathology centres are organised at different levels of complexity.

## Introduction

Although autopsy is strictly defined as dissection of a corpse in order to determine the cause of death through observation, forensic, medico-legal or judicial autopsy can have a broader definition when following a systematic approach to the macro or microscopic pathological changes that diseases produce in human beings [1]. “Medico-legal investigation of death” [2] was first spoken about back in the 1970s, going beyond the mere autopsy to include specific additional and also histopathological tests. In this sense, the autopsy is considered the gold standard test in investigating death.

The cases in which forensic autopsy is carried out are determined by the judicial system of each country. In Spain, medico-legal investigation of death is carried out by forensic physicians in cases of violent death or suspected criminality [3]. Forensic medicine is represented by the Legal Medicine and Forensic Science Institutes (hereinafter IMLCF) and the National Toxicology and Forensics Institute (hereinafter INTCF) [4], an organisational system that has been profoundly restructured since national legislation introduced the figure of the IMLCF [5] with a view to ending the traditional dispersion of forensic doctors in judicial districts [6].

There are no official figures for the number of autopsies performed worldwide. IMLCF reports estimate that around 25,000 forensic examinations are performed every year in Spain (the average number of forensic examinations per number of deaths is around 6%) [7]. The costs of forensic medical investigation are covered by the justice system, and there is no official publication indicating the cost of forensic examinations. The Spanish state budget for 2021 allocated €2.014 billion to the Ministry of Justice [8], although the costs derived from medico-legal investigation are not indicated. At regional level, the overall budget for the whole justice system in Andalusia was €526 million, of a total of over €40 billion [9].

Autopsies require human and material resources that must be programmed in order to improve the demands of the judicial system. The costs involved in performing an autopsy have been studied in several countries [10–13].

However, there is no research work into autopsy costs in Spain. The aim of this study is therefore to determine the cost of performing a standard forensic examination within the Spanish judicial system, with a view to developing alternative organisational measures and so increase and optimise resources.

The diverse range of pathology services found in Spain makes comparison difficult, particularly with regard to other countries with different legal systems and ways of working, different laboratories at their disposal, different occupational setups (for both pathologists and administrative staff, full- or part-time), etc. We therefore decided to use an activity-based costing system to calculate the cost of judicial autopsies, following the principle that the main function of the activity (the autopsy) is to transform resources (material, technological, personnel, work time, etc.) into a service (in our case, to determine the cause of death and its circumstances).

One point of interest is to consider the cost of judicial autopsies, since, although it is assumed that autopsy rates have generally shown a substantial decrease in all countries over the last few decades, it is paradoxical that this occurs due to a decrease in clinical but not medico-legal autopsies. In the USA, it is striking that clinical autopsies decreased from 16.9 to 4.3% between 1972 and 2007 (35 years), while medico-legal autopsies increased from 43.6 to 55.4% [14]. It is a similar storey in the UK, where clinical autopsy rates plummeted from 25.8% in 1979 to just 0.69% of all hospital deaths in 2013 [15]. In Spain, the decrease in the number of clinical autopsies is also evident, with the 2009 White Book of Pathological Anatomy survey showing that the average annual number of adult autopsies dropped from 22 in 2003 to 18 in 2007 [16]. This leads us to consider the estimated cost of a judicial autopsy, given the significant increase in the number of judicial autopsies in Spain, due mainly to autopsies for natural deaths which have little or no legal repercussion. One question we consider is whether the judicial system is able to support investigation of these deaths, given the cost entailed in terms of human and material resources. As a result, our starting point was to quantify the problem from a financial perspective. Our aim was to focus on a problem that affects not only the health and judicial spheres, but also the whole state administration, since the financial costs involved are significant and each government area has its own limited budget.

## Material and methods

### Type of study

Detailed cost analysis was carried out. The nominal group technique [17], which aims to pool individual opinions to reach a group decision, was applied in order to identify and quantify the resources associated with performing a forensic examination. This study was used to identify and agree on the human resources, materials and equipment associated with performing a forensic examination. The nominal group was conducted in 2 stages:

- Preparatory stage: propose resources associated with performing an autopsy, select the panel of experts and convene the nominal session.
- The nominal group consisted of ten forensic pathologists (five women and five men) with at least 15 years experience in the field; half of them worked in Andalusia and the rest in other regions, thus forming a representative group from all over the country.
- Nominal session: review the proposal drafted by the moderator, question and answer session, discussion and consensus.

### Scope of the study

Analysis was carried out from the perspective of IMLCFs in Andalusia, identifying and assessing the resources dedicated directly to forensic examinations. The resources used by other institutions in earlier stages of the investigation of a death were not considered: (a) Remove the corpse, with the attendance of the National Police/Civil Guard; (b) Attendance of medical examiner; (c) Transfer the corpse to the IMLCF; (d) Attendance of forensic teams and (e) Intervention of the Examining Court.

### Characteristics of the Spanish medico-legal research system

Spain covers a geographical area of 506,030 km^2^, with a population of 47.3 million. Andalusia has 87,268 km^2^ and a population of 8.4 million inhabitants. The total number of deaths in Spain in 2020 was 492,930, with Catalonia first with 79,685, Andalusia second with 78,160, and Madrid third with 66,583 [18]. The mortality rate in Spain was 10.38 in 2020 and 9.49 in 2021. The number of deaths in 2021 was 447,877, with Andalusia having the highest number with 79,306 [19].

### Ratios of autopsies performed in spain

In 2020, there were a total of 1133 forensic doctors in Spain, employed either by regional or state bodies. There is one forensic doctor for every 42,000 inhabitants. In 2019, approximately 25,100 forensic examinations were performed in Spain, of which 55% were for deaths from natural causes and the remaining 45% were from accidents or violence. This represents 6% of all deaths. A progressive increase in the number of autopsies is observed between 2015 and 2019, interrupted by the pandemic in 2020, and returning to the growth path in 2021.

### Stages of a forensic examination

The phases of a “standard autopsy” or model based on Recommendation (99) Three of the Committee of Ministers of the Council Europe on the methodological harmonisation of medico-legal autopsies [20] have been taken into account to calculate the costs involved in investigating the cause of death. These include all processes throughout the time the body is at the IMLCF, as shown schematically in the sections below:

- Receive and preserve the body in cold storage until the moment of the autopsy and subsequent removal by the funeral home.
- Review clinical history and information obtained when removing the body.
- External examination of the body.
- Internal examination that includes opening and studying the head, neck, thorax and abdomen.
- Additional toxicological examinations (including determining ethyl alcohol and the general system for organic compounds by enzyme immunoassay, gas chromatography-mass spectrometry) and histopathology (haematoxylin–eosin staining).

### Quantification of resources

**Fig. 1:**
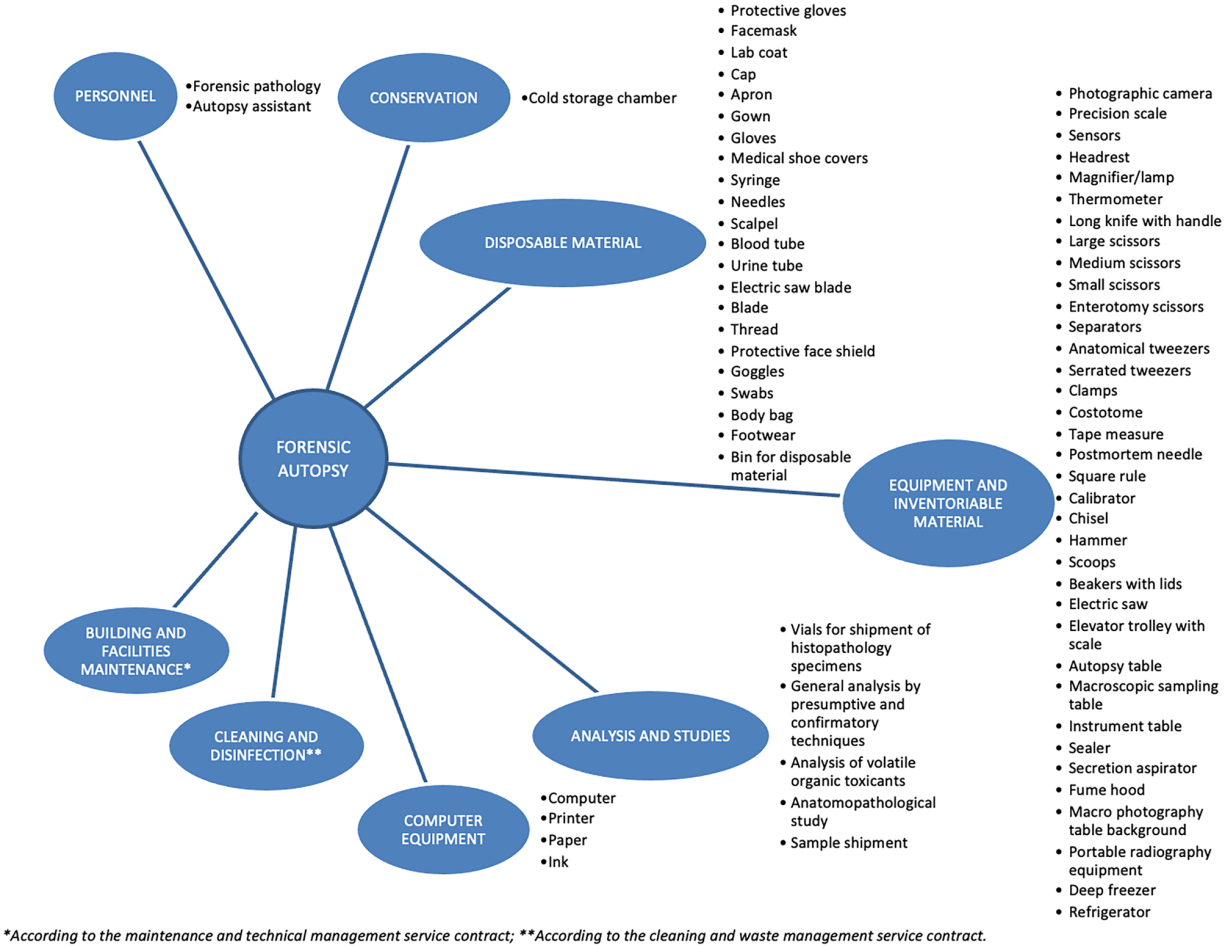
Personnel, equipment and material associated with forensic autopsy

Use of resources was estimated based on the opinion of a panel of 10 medical examiners with more than 15 years of professional experience, identifying a total of eight cost groups: personnel, single-use products, analysing and studying cases, preservation of corpse, cleaning and disinfecting, upkeep of facilities, stock and computer supplies (Fig. 1)**.**

### Assessment of costs

The resources associated with performing a standard autopsy were calculated in euros in 2021:

- Staff costs (forensic pathologists and autopsy technicians) were calculated by time-driven activity-based costing, according to official salaries [21]. The maximum annual working day, excluding vacations and public holidays, was calculated as being 1582 h, in accordance with the collective bargaining agreement.
- The cost of upkeep of facilities was calculated based on the contract for maintenance and technical management for Málaga Courthouse [22], adjusted to the scope of the IMLCF’s Forensic Pathology service: facilities, building and development work, refurbishments and improvement maintenance.
- The cost of cleaning and disinfecting was calculated in accordance with the contract for the cleaning and waste management service at the judicial facilities of the city of Malaga [23], adjusted pro rata in accordance with the floor space taken up by the IMLCF’s Forensic Pathology Service: cleaning services for facilities and fittings, environmentally friendly waste management and other additional activities.
- The costs of single-use products (autopsy material, containers for sending samples, cleaning and disinfection material) were calculated according to the market price. The number of uses was taken into account for reusable material.
- The cost of equipment and stock was calculated using the annual equivalent cost method [24], which includes the acquisition price, useful life, depreciation and cost of opportunity of the capital. The useful life was taken as 5 years, a residual value of zero and an applicable discount rate of 3%.
- Body preservation costs were calculated according to the price per day published in the Official Journal of the Province of Malaga [25].
- The cost of analysing and studying cases was estimated according to the public prices for services provided by the INTCF [26]. The cost of sending samples was also included in accordance with the cost of transport.
- Computer supplies costs were calculated according to the market price for the single-use products (paper and ink to print reports) and the annual equivalent cost method for equipment (computer and printer).

These costs are chosen as a model because they are similar throughout Andalusia, since they are awarded by public tender or published by official sources (Fig. 1).

### Statistical analysis

The cost of resources associated with performing a standard autopsy (base case) was calculated. The fixed values agreed upon by the experts were considered in order to quantify the resources, which made it impossible to calculate the values in terms of mean and standard deviation. A univariate tornado sensitivity analysis was performed in order to analyse the uncertainty of the base case results, incorporating variations in the cost components of ± 10%.

## Result

### Costs of performing a standard autopsy

**Fig. 2:**
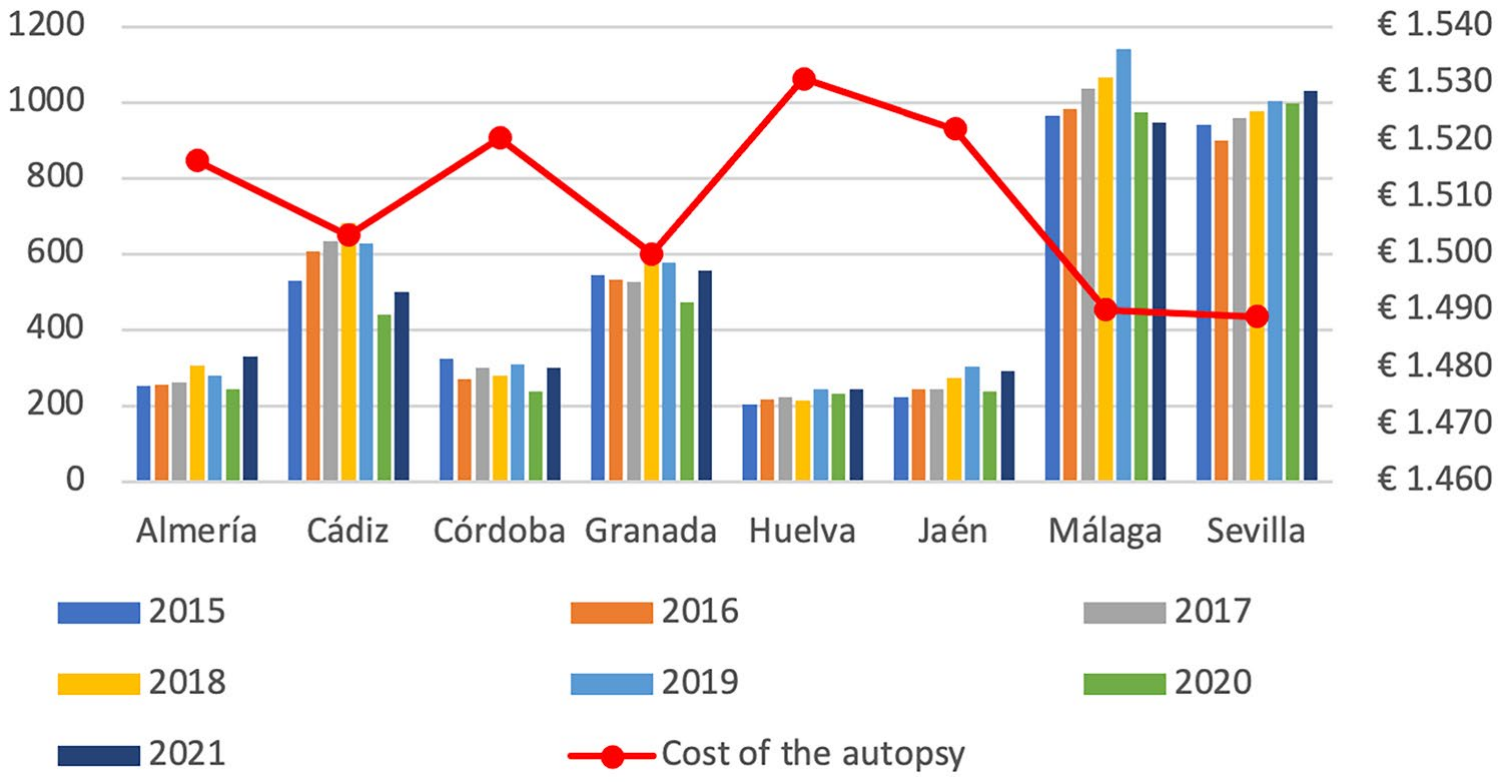
Evaluation of forensic autopsy activity and cost by province

An annual activity of 524 autopsies per centre was considered for the cost analysis, although activity levels in IMLCFs in Andalusia vary widely (Fig. 2). This figure corresponds to the average number of autopsies performed in IMLCFs in Andalusia in 2021, resulting in a figure close to the average 519 autopsies performed between 2015 and 2021. The standard cost of a forensic examination associated with the scenario defined by the experts was estimated at €1501.45.

Analysis and studies are the most important item in the total cost (54.7%), followed by personnel (20.5%), body preservation (14%), single-use products (7%), equipment and stock (1.6%), cleaning and disinfecting (1.5%), upkeep of facilities (0.5%) and computer equipment (0.2%) (Table 1)**.**

**Table 1:** Estimated cost of forensic autopsy procedure

Forensic autopsy cost items	Cost
**Personnel**	
Forensic pathology	€247.98
Autopsy assistant	€59.40
**Autopsy material**	
Disposable material	€104.82
Equipment	€24.69
**Analysis and studies**	
Sample shipment bottles	€16.68
Analysis and anatomopathological studies	€789.32
Sample shipment	€15.95
**Cadaver preservation**	
Cold storage	€209.70
**Computer equipment and reports**	
Computer and printer	€0.67
Paper and ink	€2.20
**Cleaning and disinfection**	
Clean and disinfect room	€22.87
**Maintenance of industrial buildings and facilities**	
Building and industrial facilities technician	€7.18
**Total**	**€1501.45**

### Univariate sensitivity analysis

**Fig. 3:**
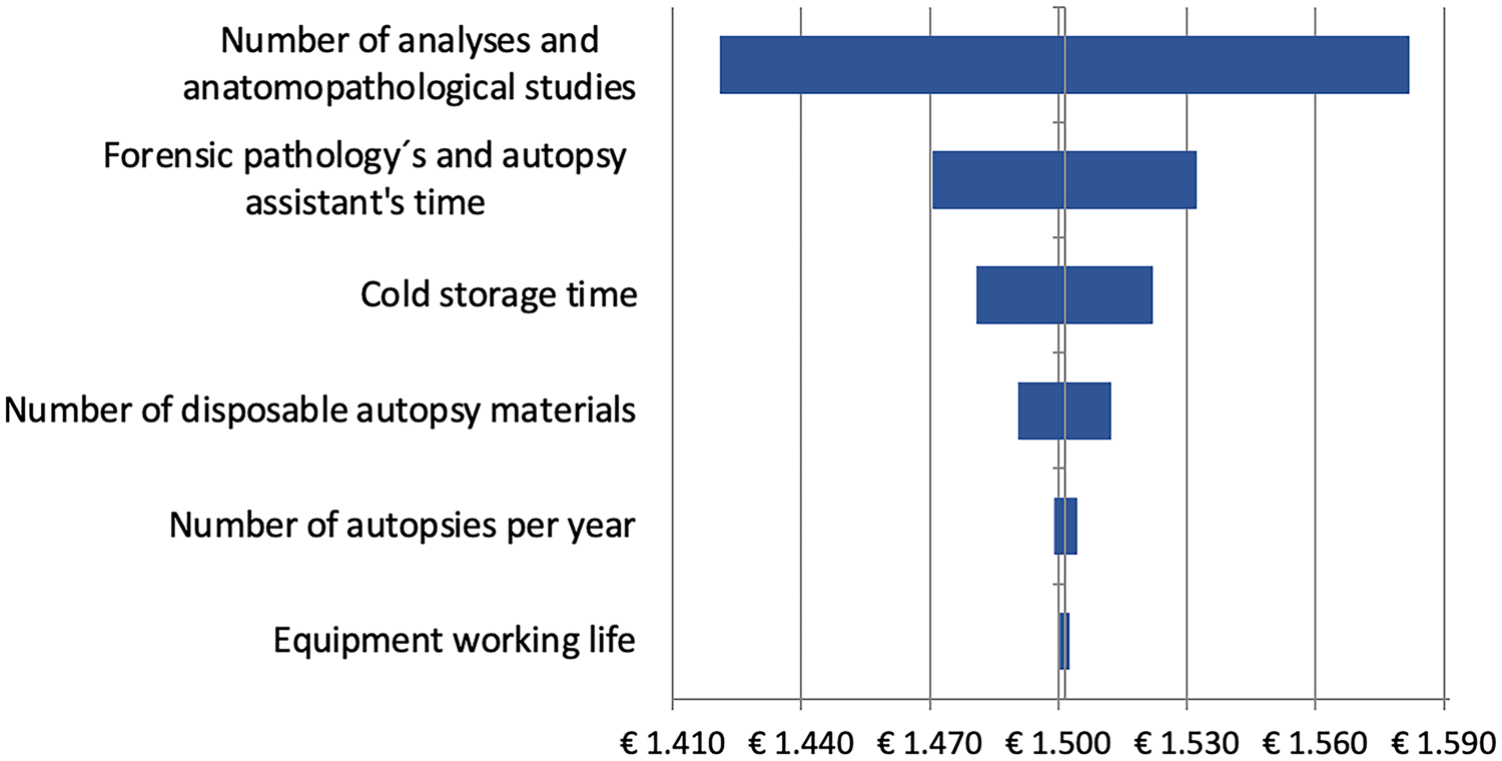
Univariate sensitivity analysis

Figure 3 shows the results of the univariate sensitivity analysis. In line with the results of the base case, it is observed that variations in the number of samples and additional studies have a greater impact on the total estimated cost, representing variations of between €1420.85 and €1582.05.

The second component with the biggest effect on the results of the base case is time spent by the forensic pathology and autopsy assistant in performing the autopsy and drafting the report, varying from €1470.71 to €1532.19.

### Results according to number of autopsies

Variations in the number of autopsies have a minor impact on the cost of the autopsy, with oscillations between €1499.14 and €1504.28, although, as can be seen in the different IMLCFs studied, significant differences are observed in terms of the number of autopsies performed in the period 2015–2021 (Fig. 2). Incorporating these activity data into the economic model gives us estimates of the cost per autopsy of between €1489 and €1531, which translates into an increase of €42 per autopsy. These differences are justified by amortisation of the equipment according to the variation in the number of autopsies performed.

## Discussion

Today it is important to ensure efficiency in allocating resources, with health, education, defence and justice all being of paramount importance. All decisions must take into account that financial resources are not unlimited. Instrumental and diagnostic techniques have been implemented over time and have also increased in the field of forensic pathology, while practise tells us that not all autopsied bodies require the full range of complementary tests available (meaning they are performed as required). We have therefore, via a detailed cost study in Spain, defined what a “standard autopsy” (a necropsy with histopathological and toxicological studies) would entail, regardless of other aspects such as social priorities, policies or legislative systems. We consider this basis to be universal in all countries of our environment since it is necessary for adequate planning of Forensic Pathology Services. A cost–benefit analysis (CBA) of investment projects is required in order to allocate available resources efficiently and effectively, whether creating or renovating Forensic Pathology Services. Costs must be taken into account when determining whether centralising services in large forensic pathology units is more effective than splitting them into smaller units and what the cost of each standard unit would be. We do not consider the quality of each Forensic Pathology Service or its regulatory requirements, but rather just the cost involved as another element for decision-making.

The total cost of a standard forensic autopsy in Andalusia has been estimated at €1501. All this is based on verified data from the detailed cost of judicial autopsies in a region that makes up 18% of the total Spanish population. Although there will logically be differences between the different Spanish regions in terms of the number of cases dealt with, Andalusia has been chosen as a representative sample since its Pathology Departments process varying numbers of autopsies throughout the region. We have studied what we call a “standard autopsy” to try to unify the real cost of an autopsy in any Forensic Pathology Service.

We compared the published costs of autopsies, first in Spain and then in the different countries, since socioeconomic conditions vary significantly, especially between countries. As far as we know, there are no publications in Spain on the cost of forensic autopsy, although there are some for clinical autopsies which can be billed by Health Services in each region. We have taken a representative sample of the Health Services in different regions with varying populations (from the lowest to the highest number of inhabitants attended to, making an estimate of the cost up to 2021—the date of our study—in accordance with the cost of living estimated in the Consumer Price Index for Spain, as shown in Table 2. It should be noted that the cost of a hospital autopsy is lower in smaller territories, increasing in larger regions from €1600 to €2300. Although some of these publications refer to the fact that the cost is established for what is called a basic clinical autopsy, we did not find the type of complementary tests included in most cases. The price calculated in our study for a forensic examination in Andalusia (€1501) is considerably lower than the price stipulated for a hospital autopsy in this region (€2300), costing just over 50% more for a clinical autopsy. The reason for this difference can be found in the low number of hospital autopsies in Andalusia, which are made more expensive by the high number of pathologists in each hospital.

**Table 2:** Cost of hospital autopsies in different Autonomous Communities in Spain, according to their Health Services and service portfolio

	Population^*^	**Publication (year)**	**Cost (€)**	**Estimated cost to year 2021 (€)**
Ceuta y Melilla [27]	85.000	2013	1.495,83	1.600
Asturias [28]	1.011.792	2009	1.231,67	1.500
Murcia [29]	1.518.486	2002	901,00	1.300
Castilla-La Mancha [30]	2.049.562	2014	1911,87	2.030
País Vasco [31]	2.213.993	2021	2.226,00	2.226
Andalucía [32]	8.472.407	2005	1.794,81	2.300
Cataluña [33]	7.763.362	2020	990,00	1.020

^*^Source: National Institute of Statistics

In other countries, several authors have researched the cost of forensic and clinical autopsy over time (Table 3)**.** Our results match those of other authors, showing a result that is practically similar to the cost estimated by authors such as Femia et al. (approximately €1500) [34], which compares conventional autopsies with others using only imaging tests (RMN/TC), without specifying how the cost of a conventional autopsy is quantified. Furthermore, our study does not include the costs of imaging tests, as these are not routinely employed during conventional autopsy, although they may be used in special cases such as gunshot wounds, charring or major trauma. Taking these tests into account would imply a significant increase in the cost of purchasing equipment, adapting and maintaining facilities and training personnel.

**Table 3:** Autopsy costs in different countries

Author	Year	Country	Cost (€)	Observations
Clark [35]	1981	USA	1.120	Clinical
Sawaguchi et al. [12]	2002	Japan	2500–2700 300–1000	Forensic Administrative
Mardiros et al. [13]	2003	Brasil	1.166	Forense Includes staff salaries
Weustink et al. [37]	2009	Netherlands	1890	Clinical Alternative MIA
Ylijoki-Sørensen [38]	2014	Finland Denmark	1400 4420	Forensic
Ahmad et al. [11]	2017	UK	548,10	Forensic
Peres [10]	2017	UK	6.900 2.300	Forensic-Paediatric Clinical-Paediatric
Gordon et al. [36]	2021	Australia	2.800	Clinical-Stillbirth
Femia et al. [34]	2021	Australia	1.500	Forensic

One of the first attempts to determine the cost of clinical autopsy is the work of Clark [35] in 1980, who estimated a cost of €1120 per case. In another study, conducted in Australia in 2021, the average cost per autopsy for foetuses that died in utero was $4200 [36] (around €2800), but this does not consider standard forensic examinations [36].

Research in the UK has studied the cost of paediatric autopsies [10] and concluded that the average cost of a paediatric forensic autopsy is around €6900, and €2300 for non-forensic cases. The disparity in the results compared to our study may be due to several reasons. Firstly, the legal system requires the intervention of a forensic paediatrician and a pathologist to perform autopsies and includes a wide range of tests such as microbiological, cytogenetic, molecular, biochemical, metabolic and toxicological analyses, which entail a considerable increase in cost. These additional studies were not included in our analysis, as they are not routinely required in the autopsy. Furthermore, our legal system does not require the intervention of two forensic doctors except in specific cases.

In contrast, according to Ahmand [11], the cost of a routine medico-legal autopsy in the UK’s National Health Service (NHS) would start at around €548,10 with a histological examination, while a virtopsy would be €262,55. A virtopsy would reduce the cost of conventional autopsy by 33%*.* The disparity in costs can only be explained by the complementary tests and the professional fees of the technicians or experts involved, and by the fact that the NHS establishes a “minimum” cost for an autopsy with a histopathological study. Given these differences, our study compares and calculates the cost of a standard autopsy based on a reduced number of complementary tests, thus allowing a range of studies to be compared.

An interesting study compares the cost of autopsies in Japan [12]. The cost of examining a body in a judicial autopsy in Japanese medical universities in 2000 was $2500–2700 (2660–2870), or $300–1000 (€320–1060) for an administrative autopsy; this is much lower than the Medical Examiner’s Office in Arkansas (USA), where each autopsy costs $6000 (€6000), while in Tokyo it would be about €4000 and in Osaka €1600. These departments carry out more duties than simply the autopsy itself (such as studying deaths without autopsies, statistics, public health), meaning their calculation may not accurately reflect the cost of an autopsy; indeed, the difference in costs is striking and highlighted by the authors in an attempt to bring judicial autopsies in Japan together in a single regulatory system. With the exception of the length of time involved, the cost of autopsy in our study is considerably lower than that reported by the State of Arkansas and Tokyo, but similar to Japanese centres where forensic examinations are performed.

Other studies do consider the items or costs of an autopsy separately (such as a study for Sao Paulo [13]^)^, breaking down items for personnel, which makes up 9.62%; the most important item is the autopsy itself (60%) followed by transportation (14%), identification and registration (14%), autopsy report (9%) and administration (3%). A cost of around $1050 (€1166) is estimated. The salaries of IMLCF staff in Spain are higher than those reported in this study.

Other reasons to calculate the cost of an autopsy is for religious reasons or due to or infection, promoting non-invasive autopsies (based solely on imaging tests) or minimally invasive autopsies (MIA, ultrasound-guided puncture for samples). Several authors, such as Weustink et al. [37] in the Netherlands, have compared the cost of MIA to conventional autopsies, but these exclude forensic cases and focus solely on clinical cases. They report that the average cost in euros per patient for MIA was 1.243,75 + /– 122,96 (with a range of 988,69–1,488.85) and 1.889,31 + /– 86,41 (with a range of 1.708,19–2.069,60) for a conventional autopsy. However, conventional autopsy, which included autopsy of the skull, was slightly higher in Weustink’s study than in ours. Although our study does not evaluate the cost of this type of autopsy, we believe that it would not substantially reduce the cost of the standard autopsy, since special equipment is necessary and a similar number of samples should be sent for additional studies, which in our case represents the most important cost item and its results are not currently applicable to all forensic cases.

ACB’s interest has been established in other European studies, such as the one carried out in Finland [38], which estimates the cost of a forensic autopsy at €1400 in Finland and at €4420 in Denmark. These authors suggest increasing the number of forensic examinations (from 2.2 to 8.5%) and medical autopsies (from 2.4 to 5.8%), which would mean an overall increase in cost but also a decrease in the unit cost from €4420 to €3094 for forensic autopsies, and from €1070 to €749 for clinical autopsies, therefore bringing an improvement in mortality statistics, public health policies and legal certainty. While the initial costs in our study are similar to those for Finland, the Finnish study does not, unlike ours, include the salaries of the forensic pathologist and autopsy technician, which could bring a variation in the final amount. However, we agree that it is essential to know the cost of a forensic autopsy, and that increasing the number of autopsies in certain centres would bring a decrease in this cost. If we take a standard forensic autopsy as being similar in cost in the different Forensic Pathology Services, then it would be logical to think that these costs would decrease when increasing the number of autopsies in large Pathology Services. Should social or regulatory policies require small Forensic Pathology Services to be maintained, the cost would become unaffordable whenever the complementary tests go beyond merely histopathological or toxicological; the need for imaging (MR, CT) or genetic tests would become more and more imperative, and the price of implementation is much higher than a “standard autopsy”, both in terms of equipment and also staff training and availability. One solution is to centralise or concentrate forensic examinations, or at least certain autopsies, in the same centre to ensure a good cost–benefit ratio. This is an initial step in quantifying the cost of a standard autopsy. However, it is well known that the cost of an autopsy depends to a large extent on the complementary tests performed [39]. Our calculation only takes into account routine toxicology and histopathology tests, such that the cost of a non-standard autopsy may increase substantially when using techniques such as immunohistochemistry, biochemistry, biology, molecular autopsy, imaging tests (MR, CT), criminalistics or microbiology. Centralising at least those autopsies that require more specific techniques and greater staff training would therefore be desirable.

Forensic Pathology Services should be organised in a pyramid structure, with basic centres for standard autopsy at the base and leading centres combining more sophisticated tests with qualified staff on the next level. This organisation would reduce autopsy costs in the long term, with the option to implement investment policies and improvements in the centres.

Our study has several limitations, such as high variability of facilities and staff between IMLCFs in Spain. Having a large number of Forensic Pathology Services could have a negative impact in terms of providing adequate facilities and staff in autopsy rooms for the different services created. Centralising them, at least for autopsies that require more sophisticated complementary tests, could increase investment and improve upkeep of facilities. Moreover, forensic pathologists in Spain are salaried personnel. However, in other countries, external staff are hired on a pay-per-act basis, which could mean the total cost of autopsies needs to be readjusted [40].

## Conclusions

This study is the first in Spain to calculate the unit price of a forensic autopsy using detailed cost analysis. Our results show that a standard autopsy costs a total of €1501.45, which is lower than in the rest of Europe. Having limited resources means the cost of an autopsy is a key aspect when determining an adequate cost–benefit ratio and ensuring public resources are well distributed. Since complementary tests are the most expensive item (55%), we believe Forensic Pathology Services should be planned at different levels of complexity in order to decrease autopsy costs and improve investment.

## Key points

1. Accurate knowledge of the cost of an autopsy could enable the development of organizational measures and strategy plans for better efficiency of public and private resources.
2. This study aimed to estimate the cost of a standard autopsy in Spain through micro-cost analysis.
3. Autopsy analysis and complementary studies represented 54.7% of the total cost.
4. The total cost of a standard autopsy was €1501.45, which is lower than the European average.
5. This study is the first in Spain to calculate the unit price of a forensic autopsy in Spain.
